# A realist evaluation of a regional Dementia Health Literacy Project

**DOI:** 10.1111/hex.12862

**Published:** 2019-01-02

**Authors:** Sandra Grace, Louise Horstmanshof

**Affiliations:** ^1^ School of Health and Human Sciences Southern Cross University Lismore New South Wales Australia

**Keywords:** Consumer health information, dementia, health literacy, patient education, realist evaluation

## Abstract

**Background:**

A Dementia Health Literacy Project was undertaken in the north coast region of NSW, Australia, after it was identified as having a high prevalence of dementia. A Dementia Support Kit was produced with service user engagement to provide useful information to people with dementia and their families.

**Objective:**

To evaluate the Dementia Health Literacy Project using a realist evaluation framework.

**Setting and participants:**

The setting was the region of the north coast of New South Wales. Eight people diagnosed with dementia and their carers, 13 members of social groups of older people in the local area, and 22 local GPs and other health‐care and service providers participated in this study.

**Results:**

Two context‐mechanism‐outcome configurations were identified: (a) co‐design workshops where the stakeholders’ opinions were equally valued (context) led service users to feel listened to and prompted them to provide feedback (mechanism) to develop a practical resource that they would use (outcome); and (b) use of health professionals to distribute the resources (context) that they consider useful and valuable (mechanism) resulted in the target audience receiving the resources (outcome).

**Discussion and conclusions:**

The Dementia Health Literacy Project produced a Dementia Support Kit that is likely to provide locally relevant and useful information for people with dementia and their carers. The results highlight the value of the co‐design approach in producing and disseminating dementia health literacy resources. Further evaluation is required to confirm the impact of the Kit over time on service users’ behaviour and consequently on their health outcomes.

## 1 INTRODUCTION

### 1.1 Background

Dementia is a common, complex and disabling disease, and many people have early cognitive decline that is not recognized or diagnosed.^1^ There are more than 400 000 Australians living with dementia and this number is expected to increase by 90% over the next 20 years.^2^ In the north coast region of NSW, Australia, 20% of residents are aged over 65 years, and almost 1 in 10 people over 65 years have dementia.^3^ This region has been classified as a region with a high prevalence of dementia with more than 1040 individual cases of dementia.^4^ Consequently, a Dementia Health Literacy Project was undertaken by the Primary Health Network in the region.

### 1.2 Dementia and health literacy

Health literacy is a measure of how well a person can find, understand and use health information. According to the Australian Commission of Safety and Quality in Health Care, health literacy includes an understanding of the “systems, processes, people, information and practices that make up the health and health care industries and sector.”^5^ Approximately 60% of the Australian adult population are at risk of low health literacy^6^ and it is a particular concern among older adults. Surveys in North America found that over 70% of adults aged older than 65 years did not have sufficient health literacy skills and were therefore unable to interact successfully with the health‐care system at a time of their lives when they were increasingly dependent on it.^7^ Low health literacy is associated with a number of poor health outcomes, including increased rates of chronic illness, early mortality, decreased use of health services and increased costs associated with health care.^8^ It is also a risk factor for hospital admission among elderly people.^8^ This is true regardless of people's cognitive performance, newspaper reading frequency, health status or level of vision.^9^ A study by Clark et al^10^ examined perceptions of self‐management and ageing among two groups of older adults: one comprised socioeconomically vulnerable adults and the other were a group of privately insured adults. The vulnerable group saw self‐management as keeping doctor visits and taking prescription medications while these were just two of many examples given by the non‐vulnerable group. Moreover, the vulnerable group did not have expectations of healthy ageing and were only able to identify a few examples of healthy ageing. In contrast, the privately insured group expressed health promotion as the key to healthy ageing. They gave many examples and had expectations of living long and healthfully into old age.

People with dementia are vulnerable to low health literacy. Many older adults are fearful of the disease and try to distance themselves from it.^11^ Corner and Bond^12^ found that fear of the condition resulted in older adults failing to seek information from health professionals. In dementia, low health literacy is associated with poor advance care planning and poor health outcomes in general.^13^, ^14^ This occurs because low health literacy affects how people access and use health care. It also affects relationships between patients and their health‐care teams, and the ability of a person to self‐manage their condition.^15^

### 1.3 Strategies to build health literacy among people with dementia, their families and carers

People with dementia report receiving little or no information, or unclear printed information about their condition and the services that are available to them.^16^ Using evidence‐based health literacy strategies can support people with dementia, their families and carers to access, understand and act on the information and services they need.^17^ Health literacy best practice has been described as: (a) information that meets health literacy standards, (b) information that is available in a variety of ways and (c) service user involvement:^6^

#### 1.3.1 Information that meets health literacy standards

Health literacy standards include using plain language, consistent font size, left justified text, chunking text into short paragraphs containing only the most important information, and using pictures rather than text where possible.

#### 1.3.2 Information available in a variety of ways

Health information needs to be delivered in a variety of ways to accommodate a variety of needs.^18^ This includes clear written information, visual aids, video decision support tools and online or digital information.^13^, ^14^, ^19^ Providing health information by video has also been shown to be an influential medium.^13^, ^14^

#### 1.3.3 Service user involvement

Person‐centred care involves partnering with patients and their carers to explore options and select the most appropriate care pathways, to understand the emotional journeys that people experience along their care pathways, and working together to improve these experiences.^20^ Partnering with patients and their carers has been shown to improve clinical quality and outcomes, people's experience of care and the business and operations of delivering care.^6^ Co‐designing health information with service users is likely to produce resources that are readily usable by the target population.

It is recommended that clinicians, health system planners and policy makers promote the uptake of these strategies into routine care to improve health outcomes for all patients, including those at risk of low health literacy. The 2016 North Coast Primary Health Network's (NCPHN) annual Needs Assessment found that access to local services and support was limited by service users’ fragmented understanding of available resources. In particular, commissioned dementia services were required in the area. Consequently, service mapping and health literacy were prioritized. A Dementia Health Literacy Project was undertaken to develop a Dementia Support Kit that would meet health literacy best practice standards. Consultations with an industry‐specific advisory group comprising clinical advisors, representatives of government agencies and non‐government partners working in the area of aged care and dementia suggested that there was a fragmented understanding of locally available services which was impacting on the ability of local services to work together in a systematic and coordinated way. The advisory group reported that those who access services do so late in their disease trajectory, when cognitive impairment is already making it difficult to make informed decisions about proactive planning and self‐management.

Toolkits have become a popular method of disseminating health information in attempts to promote positive health behaviours. A review of 83 toolkits that were designed to inform and change public and health provider behaviour by Barac et al^21^ concluded that for toolkits to be effective they needed comprehensive descriptions of evidence‐based content and they needed to be evaluated for their clinical and implementation outcomes. This was confirmed by Yamada et al^22^ who also argued for the inclusion of theory‐based content to enhance effectiveness.

### 1.4 The dementia health literacy project

The Dementia Health Literacy Project was undertaken in the north coast region of NSW, Australia, after it was identified as having a high prevalence of dementia. The Dementia Health Literacy Project adopted an experience‐based co‐design approach to design a Dementia Support Kit to provide useful information to people with dementia and their families and carers. Engaging those with the lived experience of dementia is consistent with the contemporary trend towards co‐design and co‐production of resources for health‐care quality improvement.^23^ This co‐design approach is a collaborative “method of designing better experiences for patients, carers and staff”^24^ by engaging communities, service providers and designers to solve real‐world problems.^25^ The aim is to work towards, and test, solutions with groups of people who will be directly impacted by these solutions. One of the many benefits of co‐design is improved satisfaction because of a better fit between the users and the solutions.^26^ Patients can also provide novel solutions that are relevant to their issues because they may be less rigid in their thinking compared to the professionals, although professionals’ solutions may be more technologically viable.^27^ In any case, the combination of diverse cognitive approaches and different knowledge sets promotes new ideas or the adoption of old ones in new contexts.^28^ Co‐design challenges include overcoming power imbalances and variations in commitment,^29^ allocating sufficient time and resources to co‐design, clarifying what is actually negotiable^30^ and the nature and timing of service user demands.^26^

This paper reports an evaluation of the Dementia Health Literacy Project using a realist evaluation framework. Ethics approval was provided by Southern Cross University Human Ethics Research Committee (Approval number: ECN‐17‐064). The RAMESIS II guidelines have been used to inform this report.^31^

## 2 METHOD

Realist evaluation is a way to understand how programmes work. There is an underlying assumption that no programme works for everyone all the time and that the context in which the programme occurs influences its outcome.^28^ It aims to investigate how and why an intervention works, for whom, to what extent, in which respects, in what circumstances and over what duration.^32^ That is, it aims to understand the underlying causal processes or mechanisms that generate particular behaviours and how people adapt to them. This is achieved by distinguishing salient contexts that are more or less conducive to producing the types of behaviours or adaptations of interest (outcomes). Outcomes include short‐, medium‐ and long‐term changes, both intended and unintended, that arise from an intervention.^28^ In realist evaluation, context‐mechanism‐outcome configurations are identified and can be used to explain outcome pattern variations.^33^ It was an appropriate approach for evaluating the Dementia Health Literacy Project because it could provide an understanding of how variations in mechanism and context influenced outcomes, and how those variations could be managed to improve the dementia health literacy of the target population. Realist evaluation begins with a hypothesis that can be generated from a number of sources, including literature reviews and data from large‐scale surveys. In this research, a literature review was used to generate the following hypothesis:

Engaging service users to co‐design health resources that meet health literacy standards increases health literacy, meets community needs and empowers people to feel in control of their health, to access health services and to make informed, shared decisions about their care.

### 2.1 Data collection

In realist evaluation, no strategy is ruled out in testing the hypothesis. In fact, the accumulation produced by different methods can strengthen the results. Three data collection strategies were used in this study (see Table 1):

**Table 1:** Data collection

*Stage 1: Co‐design workshops*
11 participants (8 people with dementia and their carers, 1 service provider, 2 project officers)
*Stage 2: Pilot and evaluation*
Service users
HLQ surveys
100 surveys distributed as part of Ophelia project
24 pre‐surveys returned
13 post‐surveys returned
Semi‐structured interviews
7 interviews with participants in co‐design workshops
Health‐care and service providers
Clinicians’ Survey
Invited: 24 clinicians and service providers
22 surveys returned

#### 2.1.1 Surveys

The Health Literacy Questionnaire (HLQ)^34^ was distributed to 100 older people at social groups in the local community; 24 people returned surveys before they had reviewed the Dementia Support Kit and 13 post‐questionnaires were completed. A survey was also distributed to 24 clinicians to collect feedback on the Dementia Support Kit and 22 responses were returned.

#### 2.1.2 Documentary analysis

A number of documents were collated including a health literacy literature review, the Senior Project Officer's project summary, the Dementia Support Kit and emails and meeting notes between the Health Literacy Project Officer and the research team.

#### 2.1.3 Semi‐structured interviews

Semi‐structured interviews were conducted with seven service users who had previously participated in the co‐design workshops. Three were conducted face‐to‐face; the remaining four were conducted by telephone. Interviews lasted between 30 and 60* *minutes each.

The Dementia Health Literacy Project set out to develop a Dementia Support Kit for the Tweed area. It was conducted in two stages: (a) designing a Dementia Support Kit, and (b) piloting and evaluating the Kit.

#### 2.1.4 Stage 1: Designing a dementia support kit

The Health Literacy Project Officer and the Project Officer facilitated two co‐design workshops. Invitations were sent to those who indicated their interest through responses to surveys of service users via community social groups (eg, Dementia Outreach Service), local council staff, NCPHN staff and Dementia Outreach Service workers. The agenda for the workshops was to discuss the preferred format of a regionally‐specific Dementia Support Kit, including size, length, binding and useability, the name of the resources, the layout of the resources and their content, including subjects, headings and important contacts. Co‐design workshop members were given brochures, flyers and magnets of different sizes, shapes and colours to prompt their thinking.

#### 2.1.5 Stage 2: Piloting and evaluating the dementia support kit

##### GPs and other health‐care and service providers

Copies of the Dementia Support Kit were distributed to 24 clinicians and service providers via a local clinical society event and a regional aged care symposium. A survey (the Clinician's Survey) designed to gather feedback about the usefulness of the Kit was distributed at the same time.

##### Service users with and without dementia

Service users participated in semi‐structured interviews and provided survey responses. Invitations for interviews were sent to the eight people with dementia and their carers who had participated in a co‐design workshop and seven agreed to participate. The Health Literacy Questionnaire (HLQ)^35^ was selected as a validated health literacy tool that could be used to assess change in health literacy following use of the Dementia Support Kit. The HLQ is part of the wider Ophelia project^34^ but used in this project as an evaluation tool rather than a needs assessment. It has nine domains of health literacy:

Domain 1: Health‐care provider support
Domain 2: Having sufficient information to manage my health
Domain 3: Actively managing my health
Domain 4: Social support for health
Domain 5: Appraisal of health information
Domain 6: Ability to actively engage with health‐care providers
Domain 7: Navigating the health system
Domain 8: Ability to find good health information
Domain 9: Understand health information well enough to know what to do

Six extra questions were added to the HLQ to provide specific feedback about the Kit:

1. Do you find this Kit easy to read?
2. Is this information in this Kit easy to understand?
3. What is the main message of the Kit?
4. Does the information in the Kit increase your knowledge of dementia‐related services and support?
5. How likely are you to contact any of the phone numbers in this book?
6. How likely are you to look up any of the online links in the book?

The Health Literacy Project Officer contacted three community social groups of older people that were identified via the local council website. This purposive sample of three groups was recruited to run co‐design groups as part of their scheduled meetings. The Project Officer was given permission to attend one meeting of each group to introduce the Dementia Support Kit and invite participants to evaluate the Kits. Members of the first group were invited to complete the HLQ at the beginning of the session. This was followed by an introduction to the Dementia Health Literacy Project and the development of the Dementia Support Kit. Members were then invited to take a copy of the Dementia Support Kit home and to complete the HLQ again along with the six extra questions above after they had reviewed the Kit.

At the second and third groups, information about the Dementia Health Literacy Project was presented first before the HLQ was issued. Both groups were very receptive to the information. Of approximately 100 members of the three community social groups, 24 volunteered to participate in the pilot and took a copy of the Dementia Support Kit, the HLQ with the six extra questions and a reply paid envelope so that they could review the Kit at home and return their feedback. Thirteen follow‐up phone calls or emails were made within 1‐3 weeks of the meetings to repeat the HLQ and the six extra questions about the usability of the Kit. Semi‐structured interviews were conducted with those who were phoned and face‐to‐face with three members of the co‐design group.

### 2.2 Data analysis

Analysis comprised several steps. First, semi‐structured interviews were recorded and transcribed verbatim with participants’ consent. Survey data were analysed using the descriptive statistics functions of the Survey Monkey© (1999‐2018). Next, an approach adapted from the Scanning, Analysis, Response, Assessment (SARA)^36^ was used to guide the process: (a) Scanning—identifying behaviour patterns in the context of the identified problem. Transcripts, reports, emails and open‐ended responses to survey questions were analysed independently by two members of the research team through prolonged engagement through an iterative process of reading and re‐reading the texts. They sought to identify the opportunities and constraints that were available and the circumstances or people who made them available; (b) Analysis—describing the mechanisms that generated that behaviour in the context of the identified problem; (c) Response—describing the decisions that altered the mechanisms producing the problem behaviour/state of affairs. During this stage, the project team tried to identify the reasoning or circumstances that prompted specific responses; and (d) Assessment—asking if the intervention worked as intended (outcomes). Finally, the project team considered the changes in behaviour that had or had not occurred. A draft report of the findings was circulated to the Project Officer, the research assistant and senior executive of the PHN for feedback which was incorporated into the analysis.

## 3 RESULTS

### 3.1 Participants

The evaluation involved the following participant groups:

1. Those diagnosed with dementia and their families and carers (the target group). Of the 13 people (the Project Officer, one service provider and 11 people with dementia and their family or carers) participated in the workshops, eight people with dementia and their family or carer participated in the evaluation.
2. Members of community social groups of older people who had not been diagnosed with dementia. Semi‐structured interviews were conducted by phone with 13 group members.
3. GPs and other health‐care providers and services. Of the 24 surveys distributed, 22 responses were received.

### 3.2 Context‐mechanism‐outcome configurations

Two key context‐mechanism‐outcome configurations were identified in this study:

1. Co‐design workshops where the stakeholders’ opinions were equally valued (context) led service users to feel listened to and prompted them to provide feedback (mechanism) to develop a practical resource that they would use (outcome).

Co‐design workshops enabled close engagement among patients, carers and health professionals. They provided the opportunity for people with dementia and their carers to feel listened to and that their opinions were valued and taken into account in designing the Kit (context). In such an environment, participants felt comfortable providing honest feedback (mechanism). They proposed a DL size fridge magnet (with important 24‐hour contact numbers) with additional room for personal contacts/information, a large A4 size book with spiral binding for ease of handling, large print and format for easy reading. The project team had not anticipated the preferred size of the resource. Service users thought that an A4 book was less likely to get lost as it could be easily stored on a shelf. A small fridge magnet meant that important numbers could be readily accessed. Service users also wanted a pocket on the inside cover of the book but it was beyond the limits of the budget. However, the project team were able to provide an A4 Tweed Dementia Support Kit with large print in colour and a fridge magnet that satisfied the requirements for health literacy best practice and that was likely to be used by service users (outcome). The Kit was also available online. The final Dementia Support Kit comprised the following sections (see https://ncphn.org.au/dementia):

- Important contacts
- Mind: about dementia, behaviour changes, education
- Health: general health, physical activity, going to hospital
- Living: safety, driving, younger people with dementia, aboriginal and Torres Strait Islander people with dementia
- Wellbeing: social life, feelings
- Support: help to live at home, transport, meal delivery, respite and taking a break, permanent residential care, palliative care
- Carers: planning for the future, legal matters, dying to talk
- Useful resources

Results of the HLQ showed that in Domains 2, 4, 6, 7, 8 and 9 scores increased after using the Dementia Support Kit (see Appendix S1), suggesting that the Kit had increased service users’ ability to locate, engage with, navigate and understand health information and feel more socially supported. Service users’ open‐ended comments about the Kit were generally positive, suggesting that the Kit was likely to be used (outcome). Typical responses from the six extra questions on the HLQ survey included:

It will be my “go to” for information.

Everything I need in one place.

Excellent lay‐out.

1. Use of health professionals to distribute the resources (context) that the health professionals consider useful and valuable (mechanism) resulted in the target audience receiving the resources (outcome).

A total of 22 clinicians and service providers completed the Clinicians’ Survey: nine from nursing professionals, five from service providers, three from allied health professionals, two from medical practitioners, one from a pharmacist and one from a social worker. Responses were positive about the Kit, in particular that it provided valuable and easily accessible information for service users with a diagnosis of dementia:

- 100% of respondents who reviewed the Dementia Support Kit found it easy to read and understand
- 92% of respondents thought that the Dementia Support Kit would be beneficial for their clients’ knowledge of services and support.
- 71% of respondents said that they would definitely give a hard copy of this Kit to their clients
- 35% said that they were very likely to print a copy of the Kit for their clients; 35% said that they would definitely print a copy for their clients.

Concerns raised by this group included maintaining currency of a printed resource and that printing in their office/clinic would render it black and white and less appealing to read. Vision and colour perception deteriorate with age.^37^ Consequently, sufficient colour contrast is needed to enhance readability.^38^ Concerns were also raised about accessibility to the Kit using weblinks. Although one in five people aged 65 years in the region reportedly use the Internet to look for health information online^39^ links to webpages would not be accessible to those in remote areas with poor Internet facilities. One respondent was concerned that the Kit did not accommodate culturally and linguistically diverse populations. In such cases where the health‐care professional did not rate the Kit as valuable, accessible or relevant to the target audience, then they were unlikely to distribute the resource.

Table 2 is a realist matrix summarizing the context‐mechanism‐outcome configurations at work in the Project.^40^

**Table 2:** The Dementia Health Literacy Project: Contexts, mechanisms and outcomes

Context	Mechanism	Outcome
What resources, opportunities, constraints were provided, by whom and in what circumstances?	What reasoning was prompted in response?	What changes in behaviour/state of affairs were generated?
Co‐design workshops where stakeholders’ opinions were valued. Facilitated by Project Officer, with service users diagnosed with dementia, their carers and services workers.	Service users felt listened to and were prompted to provide honest feedback	A practical resource that service users were likely to use. Produced A4 booklet and small fridge magnet containing locally relevant information.
Health‐care and service providers are well placed to distribute the Kit.	Health‐care and service providers considered the Kit to be useful and valuable Health‐care and service providers did not consider the Kit to be useful and valuable. Some had concerns about currency, the diminution of the resource if printed in black and white, and its suitability for CALD populations.	The target audience receive the Kit. The target audience do not receive the Kit.

## 4 DISCUSSION

This evaluation set out to test the hypothesis that:

Engaging service users to co‐design health resources that meet health literacy standards increases health literacy, meets community needs and empowers people to feel in control of their health, access health services and make informed, shared decisions about their care.

The Dementia Health Literacy Project brought diverse groups of people together to develop and then provide feedback on a regionally relevant Dementia Support Kit. The Project successfully delivered a resource that met health literacy best practice, used a variety of media and involved service users engagement at each stage, as recommended by the Australian Commission on Safety and Quality in Healthcare.^6^ Those who reviewed the Kit (people with dementia and their carers, older people in the local community who had not been diagnosed with dementia, clinicians and health service providers) were generally positive about it. To maximize its usefulness, it needs to be provided where it is likely to be most needed, namely to those who are diagnosing dementia and who work in dementia services. Feedback suggests that it is likely to be effective in increasing health literacy if it is appropriate to the target group (ie, those newly diagnosed with dementia and who reside in the region for which the Dementia Support Kit has been designed). In this study, many older people who had not been diagnosed with dementia did not see the relevance of the Kit (eg, covering an area where they did not live; or not relevant because they had not been diagnosed with dementia). A lack of information and misunderstanding about dementia is exacerbated by fearful older adults “attempting to psychologically distance themselves from the disease” (p. 3)^11^ which could further explain why many did not take the toolkits home with them.

It is important to use evaluation tools that are tailored to the target population. Research projects that involve people with dementia and/or carers need to take their specific needs into account (eg, response times, existence of co‐morbidities). This may involve extended timeframes for collecting feedback. For some participants, the time between the first and second rounds of the HLQ was too short for service users to provide meaningful answers to some questions. Moreover, the HLQ may not be the most appropriate tool for this target group. It is a long survey, containing 23 questions. Some questions were perceived as similar by participants. For example, participants may not have easily understood the difference between Question 4: “Feel able to discuss your healthcare concerns with a healthcare provider” and Question 20: “Ask healthcare providers questions to get the information you need.”

Project evaluation is enhanced when feedback is collected from all stakeholder groups. “Emergent” evaluation approaches, such as qualitative, participatory, empowerment and critical, all highlight the importance of stakeholder involvement in all stages of the project.^41^ In such approaches, the evaluation process is “iterative and responsive to changing circumstances and information” (p.1277). The Dementia Health Literacy Project set out to engage key stakeholders at all stages of the project. There were particular challenges associated with a stakeholder group that included people with dementia. Some could not recall being given the Kit to review or found survey questions confusing. Another challenge was the use of community groups and organizations that had no specific focus on dementia. Some health service users in these groups did not understand the relevance of the Kit for them. Evaluation was also limited by the small number of medical practitioners (one GP and one medical officer working in a hospital) who participated in the Clinicians’ Survey. The support of GPs and other health‐care and service providers is essential for wide distribution of the Kit to its target audience. Those who perform assessments and make diagnoses of dementia are best placed to distribute the Kit to the right people at the right time. Without such support access could be limited to service users’ locating the Kit themselves via Internet searches.

Ultimately, it is the target group's decisions that determine whether the desired outcome is achieved, that is, the interaction between what the Project provides and the reasoning of its intended target population that prompts the desired behaviour. This resource was intended to provide dementia health information in such a way that could influence the target group's decision making. If successful, the resource has the potential to reduce some of the negative health impacts associated with low levels of health literacy, such as increasing rates of chronic illness, decreased use of health services and increased costs associated with health care.^8^

The interventions used in this Project—co‐design workshops and a pilot and evaluation of the Dementia Support Kit—confirmed the real benefits of service user engagement at every stage of the project. The co‐design groups not only strengthened the likely impact of the final product but also made participants feel valued. The Project provided an opportunity for the Project Team to have one‐on‐one connected conversations with participants. Two causative mechanisms were identified in this evaluation:

1. When service users feel that they are listened to they are prompted to provide honest feedback. It is likely that strong service user engagement at all levels of the project will produce a resource that meets community needs and empowers people to feel in control of their health. Using location‐specific and current information (ie, information gaps in the local region that were identified in the needs analysis) can facilitate service users’ access to dementia health services in their local area.
2. When health‐care and service providers consider a resource useful and valuable, they are likely to distribute it to those who need it (ie, those diagnosed with dementia and their families and carers). Without their support, it is unlikely that the Kit would have wide distribution and uptake by the target audience. Lliffe et al,^42^ noting the inadequacy of information for people with dementia and their carers, recommended evidence‐based interventions be developed to address different stages of the disease progression. One of their studies in their *Evidence‐based Interventions in Dementia* focused on improving medical practitioners’ recognition and responses to dementia in general practice and concluded that education alone would not improve their practice. Our pilot confirmed that health providers not only need to know about the available resources, but also need to value them as sufficiently beneficial before distributing them to their patients with dementia and their carers.

### 4.1 Impact and sustainability

Further investigation is required to fully assess the impact of the Kit. Longer periods of engagement with the Kit are required before its usefulness in such areas as those described in the HLQ Domain 1 (health‐care provider support), Domain 3 (actively managing my health) and Domain 5 (appraisal of health information) can be fully evaluated. However, a co‐designed Kit that (a) meets health literacy standards, (b) provides information in a variety of ways and (c) involves service users at every stage of its development is likely to empower people to feel in control of their health, to access health services and to make informed, shared decisions about their care. Strategies to enhance the impact of the Kit included:

- Strong networks: The Health Literacy Project Officer established new relationships and re‐established pre‐existing ones with a large number of stakeholders in the aged care and dementia sectors in the region. These relationships facilitated access to targeted service users for focus groups and feedback on the Kit. Building relationships with GP Practice Nurses who can inform GPs and other medical specialists, appropriate organizations, services and service user groups may be an effective way of informing the dementia and wider community about this resource.
- Developing a dissemination plan: A sound dissemination plan was established at the commencement of the project. This included a link for clinicians to access the final version of the Kit on *Health Pathways* (a web‐based information portal supporting primary care clinicians to plan patient care through primary, community and secondary health‐care systems) and on the service user page of *Health Pathways;* a communications plan to distribute Kits to clinicians and service providers; and embedding a link to the Kit on the NCPHN website for the wider community, including a widget to the National Health Services Directory. Sustainability of any hard copy resource containing contact details of local services is limited. Dementia Support Kits for the local region require annual review of the listed services to maintain currency.
- Developing a plan to localize, produce and distribute future Kits in other regions. Regionally relevant versions are currently in development for four other regions of NSW.

## 5 LIMITATIONS

Evaluation of the Dementia Health Literacy Project was limited by the small number of service user and clinician responses that were available for analysis. Budget and time constraints limited the extent to which the usefulness of the Kit could be evaluated. The Kit needs to be disseminated more widely to those newly diagnosed with dementia and their carers and then, given adequate time to review or use the Kit, further feedback needs to be collected. Appropriate measuring tools that are delivered in a way that is sensitive to the particular needs of people with dementia and their carers are required for effective feedback of the resource.

## 6 CONCLUSION

The Dementia Health Literacy Project successfully produced a Dementia Support Kit that is likely to provide locally relevant and useful information for people with dementia and their carers. The results highlight the value of the co‐design approach in producing and disseminating dementia health literacy resources. The co‐design approach that underpinned the Project not only ensured that service users felt listened to and therefore were prompted to give honest feedback, but also ensured its usability by the target group. Health‐care and service providers need to appreciate the usefulness of the Kit to their patients and clients if they are to distribute the Kit to the target group. Further evaluation is required to confirm the impact of the Kit over time on service user behaviour and consequently on their health outcomes.

## CONFLICT OF INTEREST

The authors declare no conflicts of interest.
