# Supplementary material for: A realist evaluation of a regional Dementia Health Literacy Project
Source: Health Expect. 2019 Jan 2;22(3):426–34. doi: 10.1111/hex.12862 (PMC6543264; doi:10.1111/hex.12862)
Supplement: Supplementary file 1 [file HEX-22-426-s001.docx]

Appendix 1: Health Literacy Questionnaire (pre- and post- use of the Dementia Support Kit)

**Aim**: To evaluate the impact of the Tweed Dementia Support Kit on health literacy.

**Method**: The Tweed Dementia Kit was evaluated using the Health Literacy Questionnaire which is an Australian validated health literacy tool that measures nine scales of health literacy (Figure 1)^1^. The Kit was provided to members of various over-65s social groups in the Tweed region. Participants completed the HLQ and then took the Kits home to read and use. They were contacted again 1-3 weeks later to take the post-HLQ and answer some additional questions about the Kit itself.

*Figure 1:*


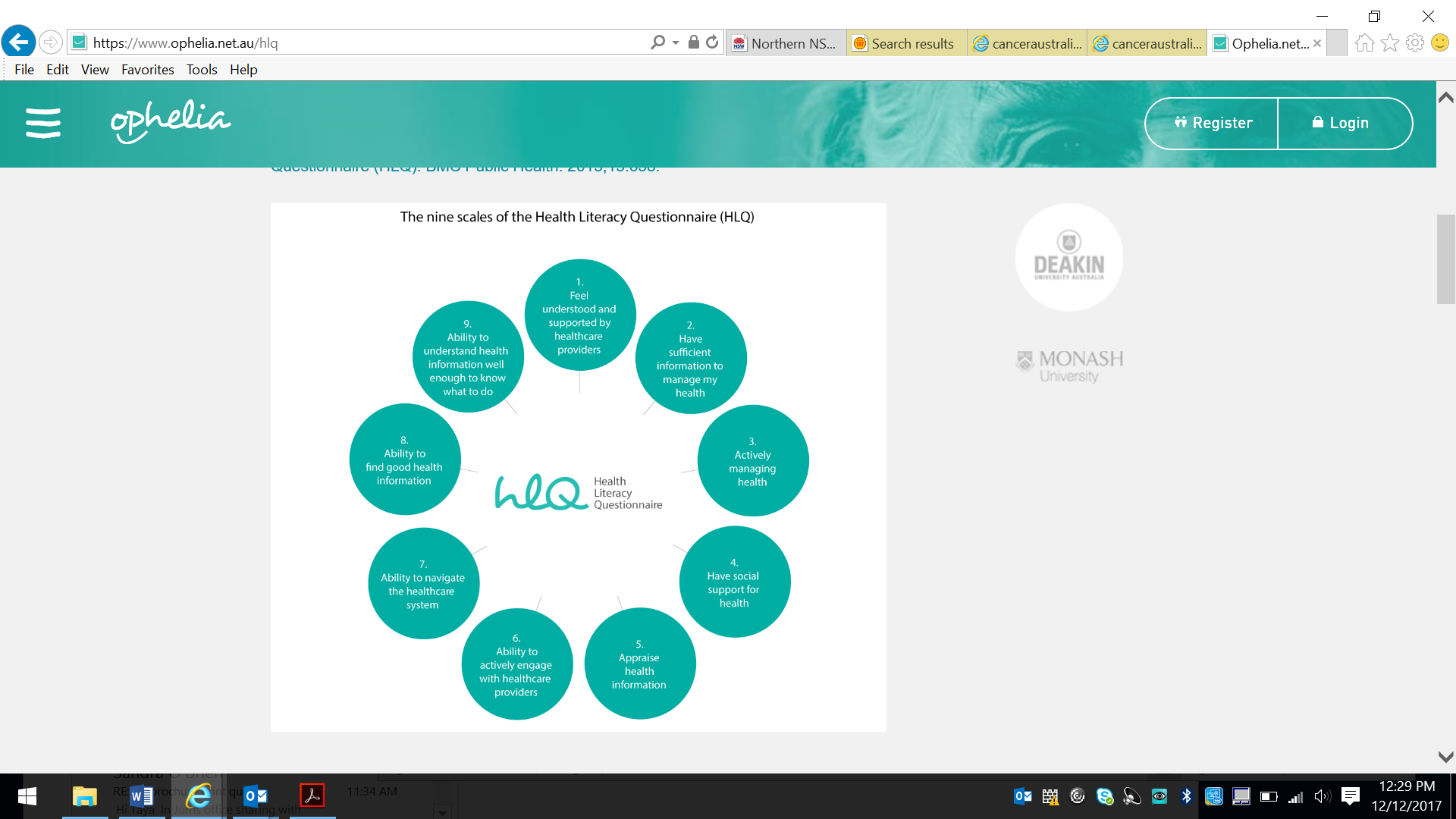


**Results**: 24 participants completed the Pre-HLQ. Follow up data was collected for 13 participants (54%), so 11 participants (46%) were lost to follow up. Results showed improvement in 6 of the 9 domains of health literacy after having the Kit for 1-3 weeks. However, due to the small sample size, the improvements were not statistically significant.

The domains in which health literacy improved were (Appendix 1):

2. Having sufficient information to manage my health

4. Social support for health

6. Ability to actively engage with healthcare providers

7. Navigating the healthcare system

8. Ability to find good health information

9. Understanding health information well enough to know what to do

The domains in which health literacy did not improve were:

1. Feeling understood and supported by healthcare providers

3. Actively managing my health

5. Appraisal of health information

**Discussion**: The Tweed Dementia Support Kit was co-designed with community and using health literacy best practice. Improvements in 2/3 of the domains, may indicate that providing clear, plain language health information designed in partnership with community members could improve health literacy. In particular, empowering people to:

- Have sufficient information to manage their health - people feel confident that they have all the information that they need to live with and manage their condition and to make decisions.
- Navigate the healthcare system - find out about services and supports so they all their health needs are met.
- Find good health information - actively use a diverse range of sources to find information and stay up to date
- Understand health information well enough to know what to do - understand written information (including numerical information) in relation to their health (Deakin, 2014)

One aim of the Kits was to support people to make decisions about their future care early in their dementia journey, while they still have capacity to do so. The improvement trend in the domain that includes decision making is a promising sign that the Kits may achieve this aim.

The results also show that the Kits may have had an impact on people feeling they had stronger social support for their health and that information about these support services has been effectively delivered. The Kits were specifically designed to provide information about the social and support services available for people with dementia, their families and carers, rather than medical services.

Finally, improvements in the domain of actively engaging with healthcare providers may indicate that the information in the Kits gave people a sense of empowerment and control over their own health. This domain indicates the Kits may support people to be proactive about their health and feel in control in relationships with healthcare providers. People are able to seek advice from additional health care providers when necessary and they are persistent in getting what they need for their health.

1. Osborne RH, Batterham R, Elsworth GR, Hawkins M, Buchbinder R. The grounded theory, psychometric development and initial validation of the Health Literacy Questionnaire (HLQ). BMC Public Health. 2013;13:658.
